# Bionic Nanoparticle Hydrogel for Astrocyte-Targeted Estradiol Delivery Ameliorates Perimenopausal Depression via P2X7 Receptor Inhibition

**DOI:** 10.34133/bmr.0336

**Published:** 2026-03-13

**Authors:** Ketan Chu, Peiqiong Chen, Wenxian Xu, Chengya Zhu, Li Xu, Chunming Li, Fan Wu, Huiyu Fan, Xiaoling Zheng, Jianhong Zhou

**Affiliations:** ^1^ Department of Gynecology, Women’s Hospital, School of Medicine, Zhejiang University, Hangzhou 310006, Zhejiang Province, People’s Republic of China.; ^2^ Department of Ultrasound, Women’s Hospital, School of Medicine, Zhejiang University, Hangzhou 310006, Zhejiang Province, People’s Republic of China.; ^3^ Department of Pharmacy, The First Affiliated Hospital of Zhejiang Chinese Medical University, Hangzhou 310006, Zhejiang Province, People’s Republic of China.; ^4^ Department of Pharmacy, Women’s Hospital, School of Medicine, Zhejiang University, Hangzhou 310006, Zhejiang Province, People’s Republic of China.

## Abstract

Estrogen (E2) therapy has shown efficacy in alleviating perimenopausal depression but is often accompanied by adverse effects when administered systemically. In this study, we developed a biomimetic nanoliposome hydrogel nasal delivery system for targeted E2 delivery and investigated its mechanisms. The system was constructed using aminated mesoporous silica as a carrier to load E2, followed by coating with astrocyte cell membranes to achieve targeting capability. The resulting nanoparticles were then embedded in a thermosensitive hydrogel composed of carboxymethyl chitosan and Pluronic F127, forming a nasal delivery platform (AEC@CP) for efficient brain targeting. A perimenopausal depression mouse model (OVX-CUMS) was established to evaluate E2’s effects on depressive behavior, hippocampal pyroptosis, and the P2X7 receptor (P2X7R)/NLRP3/caspase-1/gasdermin (GSDMD) pathway. In vitro, primary astrocytes were induced with lipopolysaccharide–adenosine triphosphate to construct a P2X7R overexpression model. AEC@CP was characterized and tested for therapeutic efficacy both in vitro and in vivo. Results showed that OVX-CUMS mice exhibited elevated astrocytic pyroptosis, which was attenuated by E2. E2 down-regulated interleukin-18 (IL-18) and IL-1β in the hippocampus and inhibited P2X7R/NLRP3/caspase-1/GSDMD signaling. In vitro, E2 suppressed astrocytic pyroptosis and associated pathway activation, and P2X7R overexpression reversed these effects. AEC@CP demonstrated excellent targeting and cellular uptake, and exerted stronger antidepressant and anti-pyroptotic effects than did free E2. In conclusion, AEC@CP effectively delivers E2 to astrocytes, suppressing the P2X7R-mediated inflammasome pathway, reducing pyroptosis, and improving depressive behavior in perimenopausal mice.

## Introduction

Perimenopausal transition, as a crucial phase in the female reproductive aging process, is closely associated with susceptibility to depressive symptoms and the development of severe depression [1]. The prevalence of depressive disorders during this particular physiological period is notably higher than in the premenopausal and postmenopausal stages [2]. Depressive symptoms during the perimenopausal period are primarily characterized by features such as sleep disturbances, emotional fluctuations, and even an increasing risk of suicide among patients [3]. Studies have indicated that estrogen (E2) deficiency resulting from ovarian function declines during the perimenopausal period and plays a key role in the pathophysiological mechanisms of severe depression associated with the perimenopausal phase [4–6]. Currently, the clinical treatment of perimenopausal depression mainly involves a treatment regimen combining antidepressants with E2 replacement therapy [7,8]. Although research has demonstrated the positive effects of E2 therapy in improving perimenopausal depression, its clinical application still faces numerous challenges due to a range of adverse reactions triggered by E2 treatment, including abnormal uterine bleeding and increased risk of thrombotic disease [9–11]. These safety issues are mainly due to unbound E2 being unable to target specific organs and causing significant hormonal fluctuations in the circulatory system. [12]. Therefore, the development of a stable and safer E2 targeted delivery system has become a key focus of current research.

Abundant studies have indicated a strong association between astrocytes and the development of depression [13–15]. Leng et al. [16] and Qi et al. [17] separately discovered a reduction in the number of astrocytes in animal models of depression and in patients with depression. In antidepressant therapy, there is an increase in the expression levels of astrocyte-related genes and a greater abundance of astrocytes [18,19]. Numerous studies have shown that pyroptosis is activated in various central nervous system diseases, including depression [20–23]. Particularly, pyroptosis mediated by the NLRP3/caspase-1/GSDMD pathway plays a critical role in a mouse model of depression [24]. Cell pyroptosis is a form of programmed cell death mediated by the gasdermin (GSDM) protein family. Caspases cleave GSDMD via the activated NLRP3 signaling pathway, leading to the rapid translocation of the N-terminal domain fragment to the plasma membrane, where it assembles into functional pores, ultimately triggering cell pyroptosis [25,26]. Furthermore, P2X7 receptor (P2X7R), as an upstream effector of NLRP3, is a key factor in activating NLRP3 and inducing cell pyroptosis [27,28]. It is noteworthy that estradiol (E2) can reverse the increased expression of P2X7R induced by ovariectomy and has an anti-pyroptotic effect in various cells, although the role of E2 in astrocyte pyroptosis remains unknown [29,30]. This suggests that targeted delivery of E2 to astrocytes to inhibit astrocyte pyroptosis may be a promising strategy for treating perimenopausal depression.

Recently, biomimetic nanocarriers with cell membrane coatings have emerged as a novel platform for targeted drug delivery. By encapsulating natural cell membranes on the surface of artificially synthesized nanoparticles, these nanocarriers can not only target specific cells but also evade immune clearance, thereby maximizing drug delivery efficiency [31–35]. Therefore, this study aims to design and develop a biomimetic nanodelivery platform using astrocyte cell membranes as coatings to precisely and effectively deliver E2 to astrocytes. However, this delivery process may still face obstacles such as the blood–brain barrier and the complex physiological environment of the human body. To address this, we have introduced a synergistic strategy involving intranasal administration combined with thermo-sensitive hydrogels. Thermo-sensitive hydrogels can undergo a sol–gel phase transition in response to the nasal cavity environment stimulation, and their 3-dimensional network structure can both load nanocarriers and prolong mucosal retention time [31–35].

Based on this, the study proposes using biocompatible aminated mesoporous silica (AMS) as a carrier to load E2, followed by coating its surface with astrocyte cell membranes to endow it with targeting capability toward astrocytes. Finally, this nanoplatform will be embedded into a thermo-sensitive hydrogel system composed of carboxymethyl chitosan (CMCS) and Poloxamer 407 (P407), forming an E2 delivery system named AMS/E2-AC@CMCS/P407 (abbreviated as AEC@CP), suitable for intranasal administration. Through intranasal delivery, this system can effectively bypass the blood–brain barrier and achieve targeted delivery of E2 to astrocytes, thereby alleviating astrocyte inflammation and pyroptosis by modulating the NLRP3/caspase-1/GSDMD pathway.

## Materials and Methods

### Construction and treatment of animal models

Ninety-three specific pathogen-free (SPF) female C57/BL mice (20 ± 2 g) were obtained from Sibeifu. Following a 1-week acclimation period under constant temperature and humidity (22 ± 1 °C, 50% to 60%), and a 12-h light–dark cycle, the subsequent experiments were conducted.

Construction of the ovariectomy-chronic unpredictable mild stress (OVX-CUMS) model: According to previous reports, the OVX-CUMS model was established [36]. Specifically, for the OVX surgery, mice were anesthetized using pentobarbital sodium (50 mg/kg, intraperitoneal injection). Following a small incision on both sides of the dorsal area, the ovaries were ligated near the uterus and then immediately removed. The incision was then sutured. The ovaries on the other side were also removed in the same manner. Sham-operated mice underwent similar procedures without ovary removal, and all surgical mice were given a 2-week postoperative recovery period. Subsequently, as described above, mice with ovaries removed underwent 4 weeks of CUMS as described previously.

Set 1: Twelve female C57BL/6J mice were divided into Sham and OVX + CUMS groups, with 6 mice in each group. The Sham group underwent sham surgery without ovary removal, while mice in the OVX + CUMS group were subjected to the OVX-CUMS model construction as described earlier.

Set 2: Forty-five female C57BL/6J mice were divided into Sham, OVX + CUMS, OVX + CUMS + E2, OVX + CUMS + NSA (pyroptosis inhibitor), and OVX + CUMS + Vehicle groups, with *n* = 6. NSA was dissolved in 1% dimethyl sulfoxide, and Vehicle received equivalent solvent only. Starting from the onset of CUMS, E2 (30 μg/kg/d) or NSA (20 mg/kg/d) treatment was initiated, with 10 μl of solution administered into each nostril, spaced 1 min apart between nostril pairs to allow for deposition in the nasal cavity. Administration was carried out once daily for 4 weeks. The control group received intranasal administration of an equal volume of 0.9% saline.

Set 3: Thirty female C57BL/6J mice were divided into Sham, OVX + CUMS, OVX + CUMS + E2, OVX + CUMS + AC@CP, and OVX + CUMS + AEC@CP groups, with *n* = 6. Starting from the onset of CUMS, daily group-specific treatments were administered, with 10 μl of solution delivered into each nostril, spaced 1 min apart between nostril pairs for deposition in the nasal cavity. Treatment was administered once daily for 4 weeks. The dosage of E2 was 30 μg/kg/d, and the amount of AC@CP and AEC@CP was calculated based on the drug loading amount. The control group received intranasal administration of an equal volume of 0.9% saline.

### Cell culture and treatment

As mentioned earlier, primary astrocytes were obtained from the cerebral cortex of neonatal C57BL/6J mice. Astrocytes were cultured in Dulbecco’s modified Eagle’s medium/F12, supplemented with 10% fetal bovine serum and 1% penicillin/streptomycin. The culture dishes were placed in a constant temperature incubator at 37 °C with 5% CO_2_.

Sangon Biotech was commissioned to construct a P2X7R overexpression lentiviral vector, which was then transfected into astrocytes to establish astrocytes overexpressing P2X7R.

### Nissl staining

After embedding the hippocampal tissue in paraffin, it was sectioned into 4-μm-thick slices. These slices were sequentially immersed in a gradient of ethanol concentrations, followed by staining with Nissl staining solution for 20 to 40 min. After a slight rinse with distilled water, the slices were differentiated in 95% ethanol, dehydrated in absolute ethanol, and made transparent in xylene. Finally, neutral resin was used for mounting, and the samples were observed under a microscope.

### Immunohistochemistry

Hippocampal sections were deparaffinized and rehydrated through xylene and a gradient of ethanol concentrations. Tissue was incubated in blocking solution for 1 h to block nonspecific binding. Subsequently, primary antibody against glial fibrillary acidic protein (GFAP) (ab7260, Abcam) was added to the tissue and incubated overnight at 4 °C. The next day, after washing with phosphate-buffered saline (PBS), goat anti-rabbit secondary antibody (Invitrogen) was applied and incubated at room temperature for 1 h. After washing away unbound antibodies, the sections were coverslipped and observed under a microscope for imaging.

### ELISA detection of IL-18 and IL-1β levels

Following the manufacturer’s instructions, Mouse IL-18 ELISA Kit (PI553, Beyotime) and Mouse IL-1β ELISA Kit (PI301, Beyotime) were used to detect the levels of interleukin-18 (IL-18) and IL-1β in mouse hippocampal tissue, respectively.

### Immunofluorescence colocalization

Hippocampal sections were deparaffinized and rehydrated through xylene and a gradient of ethanol concentrations. Tissue was incubated in blocking solution for 1 h. Subsequently, GSDMD (20770-1-AP, Proteintech) was added to the tissue and incubated overnight at 4 °C. The next day, after washing away unbound GSDMD with PBS, fluorescent secondary antibodies were applied and incubated at room temperature for 1 h. After washing away excess secondary antibodies with PBS, IBA1 (ab178846, Abcam) or GFAP (16825-1-AP, Proteintech) was added and incubated overnight at 4 °C. On the third day, after washing away unbound antibodies with PBS, fluorescent secondary antibodies were applied and incubated for 1 h, followed by PBS rinsing. 4′,6-Diamidino-2-phenylindole (DAPI) staining solution was added. After 10 min, PBS was used for washing, and anti-fade mounting medium was applied for slide preparation for observation under a fluorescence microscope.

### Sucrose preference test

Prior to the experiment, all mice were administered a 1% sucrose solution orally for 72 h, followed by a 24-h period of food and water deprivation. Each cage contained one bottle of water and one bottle of 1% sucrose solution, with the positions of the 2 bottles exchanged after 12 h.

$$\begin{array}{c} \begin{array}{c} \text{Sucrose preference}\;\left(\%\right)=[\text{Amount of sucrose consumed}/\\ \left(\text{Amount of sucrose consumed}+\text{Amount of water consumed}\right)]\times 100\% \end{array} \end{array}$$ (1)

### Forced swim test

Mice were individually placed in a transparent cylindrical water tank (diameter 20 cm, height 30 cm, water temperature 25 ± 1 °C) with a water depth of 15 cm, ensuring that the mice could not escape or touch the bottom. The initial experiment (preliminary test) lasted for 6 min, during which the immobility time (defined as the time when the animal stopped struggling and only maintained a passive state with its head above the water surface) in the following 4 min and the time when the animal first entered the immobile state were recorded. The formal experiment was conducted 24 h later, with the immobility time in the subsequent 4 min and the time when the animal first entered the immobile state recorded over a 6-min period.

### Open field test

The experiment was conducted in a 40 cm × 40 cm black organic glass cube. Mice were placed into the box from the lower left corner and allowed to explore freely for 15 min before being removed and returned to their cage. After each experiment, the test box was thoroughly cleaned with 75% alcohol for the next mouse’s use. Data including the center zone movement distance, total movement distance, and number of entries into the center zone were recorded, exported, and analyzed.

### Western blot

Total protein was extracted from mouse hippocampal tissue or astrocytes, and the protein concentration was determined. The proteins were denatured by boiling. After electrophoresis and transfer, the target proteins were transferred to a polyvinylidene fluoride (PVDF) membrane. Specific primary antibodies against NLRP3 (ab263899, Abcam), GSDMD, caspase-1 (31020-1-AP, Proteintech), or glyceraldehyde-3-phosphate dehydrogenase (GAPDH) (60004-1-Ig, Proteintech) were incubated at 4 °C. The following day, after washing with tris-buffered saline with Tween (TBST) to remove excess antibodies, corresponding secondary antibodies were applied and incubated for 1 h. After washing with TBST, an enhanced chemiluminescence substrate was added for visualization.

### Quantitative real-time PCR

Total RNA was extracted from tissues using TRIzol reagent (Thermo Fisher Scientific) according to the manufacturer’s instructions. Complementary DNA (cDNA) was synthesized using the PrimeScript RT Reagent Kit (RR047A, Takara). mRNA primers were obtained from Sangon Biotech Co. Ltd. The expression levels of target mRNAs were quantified using a quantitative real-time polymerase chain reaction (qRT-PCR) system (Bio-Rad), with GAPDH serving as the internal control. Primer sequences are provided in Table S1. Relative expression was calculated using the 2^−ΔΔCt^ method.

### DHE staining

Following the manufacturer’s instructions, the Frozen Section ROS Detection Kit (DHE probe) (HR9069, BioVision) was used to detect the levels of reactive oxygen species (ROS) in frozen sections of fresh mouse hippocampal tissue.

### ROS level measurement

The fluorescence probe 2,7-dichlorofluorescin diacetate (DCFH-DA) (S0033M, Beyotime) was added to astrocytes and incubated at 37 °C in the dark for 30 min. After washing to remove unbound probe, DAPI staining was added for 10 min. After washing away excess staining solution, anti-fade mounting medium was applied for slide preparation, and observation was done under a fluorescence microscope.

### AM/PI staining

According to the instructions of the Calcein/PI Cell Viability and Cytotoxicity Detection Kit (C2015S, Beyotime), calcein AM/PI (propidium iodide) detection working solution was added to astrocytes and incubated at 37 °C in the dark for 30 min. After incubation, observation was carried out under a fluorescence microscope.

### LDH release level measurement

Using the LDH Cytotoxicity Assay Kit (C0016, Beyotime) according to the instructions, the release level of lactate dehydrogenase (LDH) from astrocytes was measured.

### Material preparation

#### Preparation of AMS

Under room temperature conditions, 0.5 g of cetyltrimethylammonium chloride (CTAC) and 0.06 g of triethanolamine (TEA) were dissolved separately in 2 ml of deionized water and then adjusted to 10 ml to prepare solution A. Simultaneously, an appropriate amount of tetraethyl orthosilicate (TEOS) was mixed with 0.2 ml of bis[3-(triethoxysilyl)propyl] tetrasulfide (BTES) to form solution B after sonication. Solution A was placed in an 80 °C constant temperature stirrer, and solution B was slowly added dropwise with continuous stirring for 4 h. After the reaction, the mixture was centrifuged, the supernatant was discarded, and the product was washed twice. The obtained product was dispersed in 20 ml of template removal agent and subjected to 12 h of condensation reflux treatment, followed by centrifugation and washing. Finally, the precipitate was vacuum-dried at 60 °C to obtain mesoporous silica (MS) material. For amination modification, 5 mg of MS was dispersed in 10 ml of water, and 0.1 ml of (3-aminopropyl) triethoxysilane was added with continuous stirring for 8 h to obtain AMS materials.

#### Isolation of astrocyte cell membranes

Astrocytes were washed once with PBS, scraped off using a cell scraper, and mixed with isolation buffer-1 [IB-1: containing 75 mM sucrose, 225 mM mannitol, 0.5 mM acetic acid, 0.5% w/v bovine serum albumin, 30 mM tris-hydrochloride (pH 7.4), 1% v/v protease inhibitor, and 1% v/v phosphatase inhibitor mixture] before being stored on ice. Subsequently, the mixture underwent 30 min of sonication in a cold environment. The solution was then centrifuged at 800*g* for 10 min at 4 °C to remove intact cells and nuclei. The supernatant was further centrifuged at 10,000*g* for 20 min at 4 °C to remove the pellet containing mitochondria. Finally, the supernatant was centrifuged at 25,000*g* for 40 min at 4 °C to obtain astrocyte cell membranes (referred to as AC) [37].

#### Preparation of biomimetic E2 nanoparticles (AMS/E2-AC)

E2 was dispersed in a PBS solution of AMS (mass ratio of 1:10) and stirred at room temperature for 24 h to obtain E2-loaded AMS/E2 nanoparticles. Subsequently, freshly prepared astrocyte cell membrane fragments were added to AMS/E2 at a 1:1 mass ratio, and the mixture was extruded through a 220-nm polycarbonate membrane to obtain AMS/E2-AC. Further purification of AMS/E2-AC was achieved through ultrafiltration centrifugation (molecular weight cutoff: 100 kDa) [31]. The particle size of AMS/E2 and AMS/E2-AC (sample concentration of 100 μg/ml) was measured using dynamic light scattering (DLS). The microstructure of AMS/E2-AC was observed using transmission electron microscopy (TEM) (Hitachi, Japan). Specifically, 100 μg/ml AMS/E2-AC was prepared; 8 μl was placed on a carbon-coated copper grid, allowed to settle for 2 min, and stained with 1% phosphotungstic acid for 3 min; and excess liquid was removed, air-dried, and imaged. The zeta potential of AMS, AMS/E2, and AMS/E2-AC was determined using a Zetasizer Nano ZS ZEN3600 (Malvern); the protein composition of astrocyte lysate, astrocyte cell membrane, and AMS/E2-AC was analyzed using sodium dodecyl sulfate (SDS)–polyacrylamide gel electrophoresis (PAGE). The encapsulation efficiency and drug loading were calculated using the following formulae:

$$\begin{array}{c} \begin{array}{c} \text{Encapsulation efficiency}=\left(W\_\text{initial}-W\_\text{free}\right)/W\_{\text{initial}}^{*}\;100\% \end{array} \end{array}$$ (2)

$$\text{Drug loading}=\left(W\_\text{initial}-W\_\text{free}\right)/M^{*}\;100\%$$ (3)

where *M* is the total amount of AMS/E2-AC.

#### Preparation of AEC@CP

Three hundred milligrams of P407 solution was added to 5 ml of AMS/E2-AC dispersion (1 mg/ml) and dissolved overnight at 4 °C. Subsequently, 400 mg of CMCS was dispersed in an equivalent concentration and volume of AMS/E2-AC dispersion, stirred until completely dissolved. The 2 solutions were slowly mixed in an ice bath and homogeneously mixed, and AEC@CP was prepared.

#### Characterization of AEC@CP

Gelling behavior was observed at room temperature and at 34 °C water bath for 2 min, with photographs recorded. For microstructure, AEC@CP was gelled at 34 °C, frozen at -80 °C, freeze-dried for 24 h, cut into small pieces, gold-coated, and imaged via scanning electron microscopy (SEM) to observe the distribution of nanoparticles within the gel.

### In vitro study on the transdermal release behavior of AEC@CP

The in vitro release of AEC@CP was evaluated using high-performance liquid chromatography (HPLC). A drug permeation test was performed using a Franz diffusion cell. Porcine nasal mucosa was fixed between the donor and receptor compartments with the mucosal side facing the donor compartment. The receptor compartment contained 10 ml of receiving solution (1.0% SDS buffer solution) and was continuously stirred at 200 rpm at 34 ± 0.5 °C. At specific time points (1, 2, 4, 8, 12, 24, 36, 48 h), 1 ml of PBS buffer was withdrawn and replaced with an equal volume of PBS buffer. Subsequently, HPLC was used to measure the E2 content at each time point, calculate the cumulative E2 release amount (S) at each time point, and plot the in vitro drug release curve.

$$S\%=\frac{\sum_{i=1}^{n-1}V_{a}C_{i}+V_{0}C_{n}}{M}\times 100\%$$ (4)

where *V*_0_ is the total volume of the release medium (*V*_0_ = 10 ml), *V*_a_ is the sample volume (*V*_a_ = 1 ml), *C_n_* is the concentration of E2 at the *n*th sampling, and *M* is the total amount of E2.

### CCK-8 assay

After drug treatment, the medium was removed, and 10% Cell Counting Kit-8 (CCK-8) solution was added to the cells. The cells were returned to the incubator for further incubation. After 1.5 h, the cells were removed, and the absorbance of each well at 450 nm was measured.

### Cell uptake experiment and endocytosis mechanism analysis

E2 labeled with Cy5 was used to prepare AE@CP without cell membrane encapsulation and AEC@CP with cell membrane encapsulation. The uptake of these formulations by astrocytes was observed using a fluorescence microscope after 4 h.

Astrocytes and neuronal cells were incubated with fluorescently labeled AE@CP or AEC@CP at equivalent concentrations for different time intervals (1, 2, and 4 h). After incubation, cells were washed with cold PBS, harvested, and analyzed by flow cytometry to quantify nanoparticle internalization.

To investigate the endocytosis mechanisms, astrocytes were pretreated for 30 min with specific endocytosis inhibitors prior to nanoparticle incubation: amiloride (macropinocytosis inhibitor), chlorpromazine (clathrin-mediated endocytosis inhibitor), methyl-β-cyclodextrin (M-β-CD; lipid raft inhibitor), and lovastatin (caveolae-mediated endocytosis inhibitor). Cells were then incubated with AEC@CP, followed by flow cytometry analysis. Uptake efficiency was expressed as mean fluorescence intensity (MFI) and normalized to the untreated control group.

### In vivo imaging and toxicity assessment

In order to study the targeted accumulation of AEC@CP in the brain, Cy5-labeled AE@CP and AEC@CP were administered intranasally to mice. The near-infrared fluorescence intensity of Cy5 was monitored at different time intervals using a small animal in vivo imaging system. Mice were euthanized 24 h after administration, and major organs (heart, liver, spleen, lungs, kidneys, brain) were imaged. Additionally, to investigate the uptake of AE@CP and AEC@CP by astrocytes, hippocampal slices were obtained from mice, and immunofluorescent detection of GFAP in astrocytes was performed. For biosafety evaluation, blood samples were collected for serum biochemical analysis, including alanine aminotransferase (ALT), aspartate aminotransferase (AST), triglycerides (TGs), cholesterol (CHO), blood urea nitrogen (BUN), and creatine kinase (CK). Meanwhile, major organs were fixed, sectioned, and subjected to hematoxylin and eosin (H&E) staining for histopathological examination.

### Data analysis

Data processing was conducted using GraphPad Prism 8 statistical analysis software. One-way analysis of variance (ANOVA) was used to compare statistical differences among multiple groups, supplemented by Tukey’s multiple comparison test. Experimental results were presented as mean ± standard deviation (mean ± SD) from no fewer than 3 independent replicates. The statistical significance threshold was set at *P* < 0.05.

## Results

### Perimenopausal depression model mice show astrocytic pyroptosis in the hippocampal tissue

**Fig. 1.**
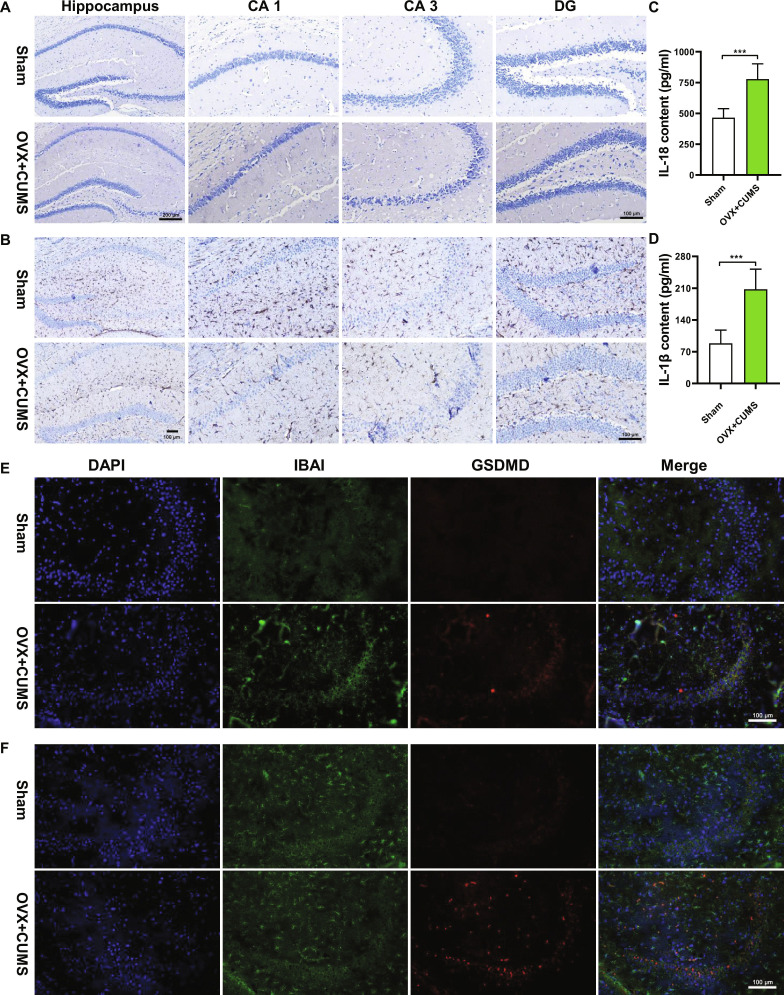
Changes in hippocampal astrocytes in a perimenopausal depression model in mice. (A) Nissl staining to detect morphological and numerical changes in hippocampal neurons. (B) Immunohistochemistry to assess the number of hippocampal astrocytes. (C and D) ELISA to measure the expression of apoptotic products IL-18 and IL-1β in the hippocampus. (E) Immunofluorescence to examine the colocalization of microglia cells and apoptotic proteins in the hippocampus. (F) Immunofluorescence to investigate the colocalization of astrocytes and apoptotic proteins in the hippocampus. ***P < 0.001.

In order to investigate the changes in astrocytes in the hippocampal tissue of mice with perimenopausal depression, an OVX + CUMS model was established. Nissl staining of the hippocampus (Fig. 1A) showed that in the Sham group, the hippocampal neurons were intact and clear, with abundant and dense blue patches visible in the cytoplasm, while the number of hippocampal neurons was significantly reduced in the OVX + CUMS group. Immunohistochemistry staining of hippocampal astrocytes (Fig. 1B) revealed that compared to the Sham group, the number and area of hippocampal astrocytes were reduced in the OVX + CUMS group.

Subsequently, enzyme-linked immunosorbent assay (ELISA) experiments were conducted to detect the expression of apoptotic products IL-1β and IL-18 in the hippocampus (Fig. 1C and D). Compared to the Sham group, the expression levels of IL-1β and IL-18 were significantly increased in the OVX + CUMS group, indicating the occurrence of cell pyroptosis in the perimenopausal depression model. Furthermore, immunofluorescence colocalization staining was used to determine the location of pyroptosis. The results in Fig. 1E showed that in the OVX + CUMS group, there was a small amount of colocalization between microglia cells (IBA1, green) and apoptotic protein (GSDMD, red). Figure 1F demonstrates that in the OVX + CUMS group, there was a significant colocalization between astrocytes in the hippocampus (GFAP, green) and pyroptosis protein (GSDMD, red), indicating that perimenopausal depression model is associated with more astrocytic cell pyroptosis.

### E2 alleviates depression behavior in perimenopausal mice

**Fig. 2.**
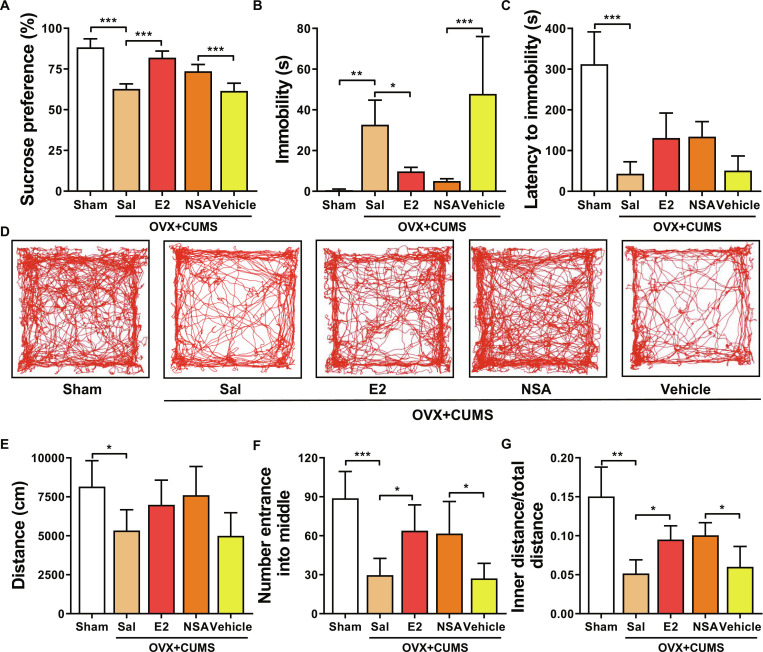
Effects of E2 on perimenopausal depressive behavior in mice. (A) Sucrose preference test. (B) Immobility time in the forced swimming test. (C) Time to first immobility in the forced swimming test. (D) Open field test mouse movement trajectory. (E) Total distance moved by mice in the open field test. (F) Number of entries into the central zone by mice in the open field test. (G) Ratio of distance moved in the central zone to total distance moved by mice in the open field test. *P < 0.05, **P < 0.01, ***P < 0.001.

To investigate the impact of E2 on depression behavior in perimenopausal mice, we treated perimenopausal depression model mice with E2. The preference for sucrose solution was measured to assess their depressive symptoms. Compared to the OVX + CUMS group, the sucrose preference of perimenopausal depression mice significantly increased after treatment with E2 or NSA, with no significant difference between the 2 (Fig. 2A). The depressive state was evaluated by observing the behavior of mice in an inescapable swimming environment. The results showed that after E2 or NSA treatment, the immobility time of mice significantly decreased, and the time to first immobility state increased (Fig. 2B and C). The depression level of perimenopausal depression mice was assessed through an open field test. The results indicated that after E2 or NSA treatment, the total distance traveled by mice showed no significant difference compared to the Sham group. Additionally, compared to the OVX + CUMS group, the number of entries into the central area and the ratio of distance traveled in the central area to the total distance significantly increased (Fig. 2D to G). These data suggest that E2 can significantly improve depressive behavior in perimenopausal depression mice.

### E2 alleviates hippocampal astrocytic pyroptosis in perimenopausal depression model mice

**Fig. 3.**
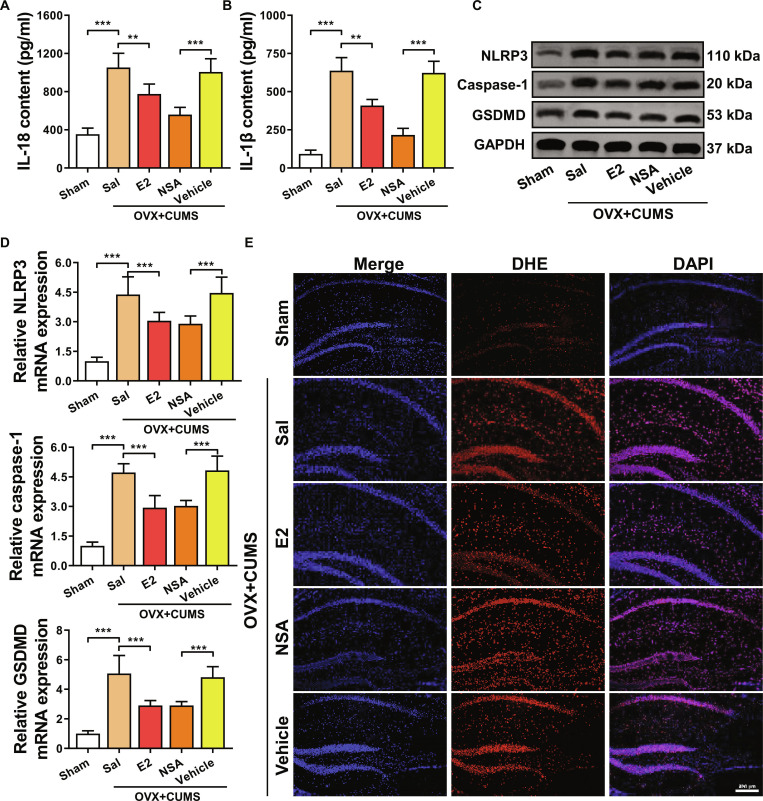
The effect of E2 on astrocytic pyroptosis in perimenopausal depression model mice. (A) ELISA assay to detect the expression of apoptotic product IL-18 in the hippocampus. (B) ELISA experiment to measure the expression of apoptotic product IL-1β in the hippocampus. (C) Western blot analysis to detect the expression of pyroptosis-related proteins. (D) Quantitative real-time PCR (qRT-PCR) analysis of pyroptosis-related gene expression. (E) Cell fluorescence method to measure the content of ROS in the hippocampal tissue. *P < 0.05, **P < 0.01, ***P < 0.001.

We further investigated the effects of E2 on hippocampal astrocytes in perimenopausal depression model mice. The results in Fig. 3A showed that the expression levels of IL-1β and IL-18 were significantly up-regulated in perimenopausal depression model mice. After treatment with E2 or NSA, the expression of apoptotic products IL-1β and IL-18 in the hippocampal tissue of perimenopausal depression model mice significantly decreased. Figure 3B demonstrates that compared to the Sham group mice, the levels of NLRP3, GSDMD, and caspase-1 in the hippocampal tissue of OVX + CUMS group mice were significantly up-regulated. However, after E2 or NSA treatment, the levels of NLRP3, GSDMD, and caspase-1 in the hippocampal tissue of OVX + CUMS group mice were significantly down-regulated (Fig. 3C and Fig. S1). These findings were further corroborated by qRT-PCR analysis (Fig. 3D). This indicates that E2 can significantly inhibit astrocytic pyroptosis in the hippocampal tissue of perimenopausal depression mice.

Further validation of the colocalization relationship between hippocampal astrocytes and GSDMD through immunofluorescence revealed a strong colocalization between astrocytes and GSDMD in the hippocampus. Moreover, the expression of GSDMD was enhanced in the OVX + CUMS group, suggesting an excessive expression of GSDMD in the perimenopausal depression model, which could be reduced by E2 or NSA in the hippocampal astrocytes of OVX + CUMS group mice (Fig. S2). Pyroptosis is typically accompanied by the extensive production of ROS [38]. Following treatment, ROS staining results in the hippocampal tissue demonstrated an increase in ROS content in perimenopausal depression model mice, resulting in severe oxidative stress. Treatment with E2 and NSA could to varying degrees inhibit the production of ROS in the hippocampal region of perimenopausal depression model mice, alleviating oxidative stress. E2 exhibited the most significant inhibitory effect on ROS production (Fig. 3E).

### E2 inhibits pyroptosis by suppressing P2X7R protein expression in astrocytes

**Fig. 4.**
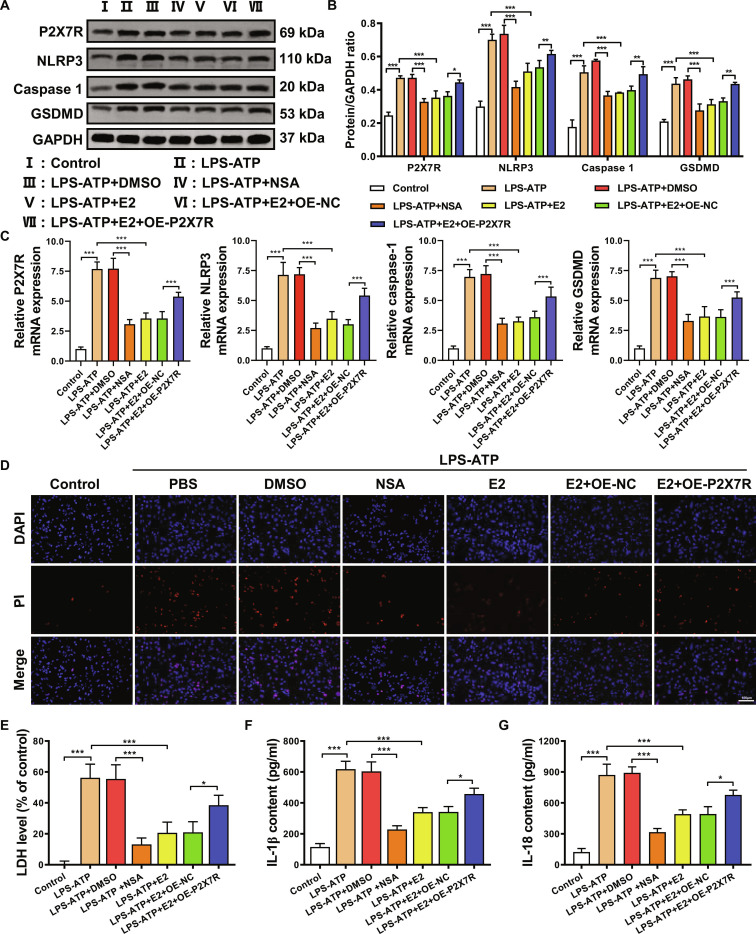
The effect of E2 on P2X7R expression in astrocytes of perimenopausal depression model mice. (A and B) Western blot analysis of P2X7R and pyroptosis-related protein expression. (C) qRT-PCR analysis of P2X7R and pyroptosis-related gene expression. (D) AM/PI staining to assess cell viability. (E) Assay kit to measure LDH release. (F and G) ELISA assay to detect the expression of apoptotic products IL-18 and IL-1β in the hippocampus. *P < 0.05, **P < 0.01, ***P < 0.001.

To investigate the mechanism by which E2 inhibits astrocytic pyroptosis, we induced a cell pyroptosis model in astrocytes using lipopolysaccharide (LPS)–adenosine triphosphate (ATP) treatment and overexpressed the P2X7R protein. Western blot results in Fig. 4A and B demonstrate that treatment with NSA or E2 significantly inhibits the expression levels of P2X7R protein and downstream NLRP3–caspase-1–GSDMD pathway proteins. Conversely, overexpression of P2X7R leads to a noticeable increase in P2X7R protein levels, along with a moderate increase in the expression levels of downstream NLRP3–caspase-1–GSDMD pathway proteins. Similarly, qRT-PCR analysis (Fig. 4C) yielded consistent results. This suggests the successful construction of astrocytes overexpressing P2X7R and indicates that E2 may alleviate astrocytic pyroptosis by inhibiting P2X7R.

Results from AM/PI staining, as shown in Fig. 4D, reveal that treatment with NSA or E2 reduces LPS-ATP-induced cell pyroptosis, with the therapeutic effect of E2 weakening upon overexpression of P2X7R. LDH release assay is a typical strategy to evaluate cell pyroptosis, as pyroptosis leads to plasma membrane rupture, resulting in rapid LDH release into the extracellular space [39]. Figure 4E illustrates that both NSA and E2 significantly reduce LDH release, indicating inhibition of cell pyroptosis. However, compared to treatment with E2 alone, overexpression of P2X7R leads to an increase in LDH release, suggesting that P2X7R overexpression promotes astrocytic pyroptosis. Figure 4F and G demonstrates that E2 and NSA treatment can decrease the expression levels of apoptotic products IL-1β and IL-18, while overexpression of P2X7R results in elevated levels of IL-1β and IL-18 compared to treatment with E2 alone. These results indicate that E2 effectively inhibits cell pyroptosis by suppressing P2X7R protein expression in astrocytes.

### Characterization of the AEC@CP drug delivery system

**Fig. 5.**
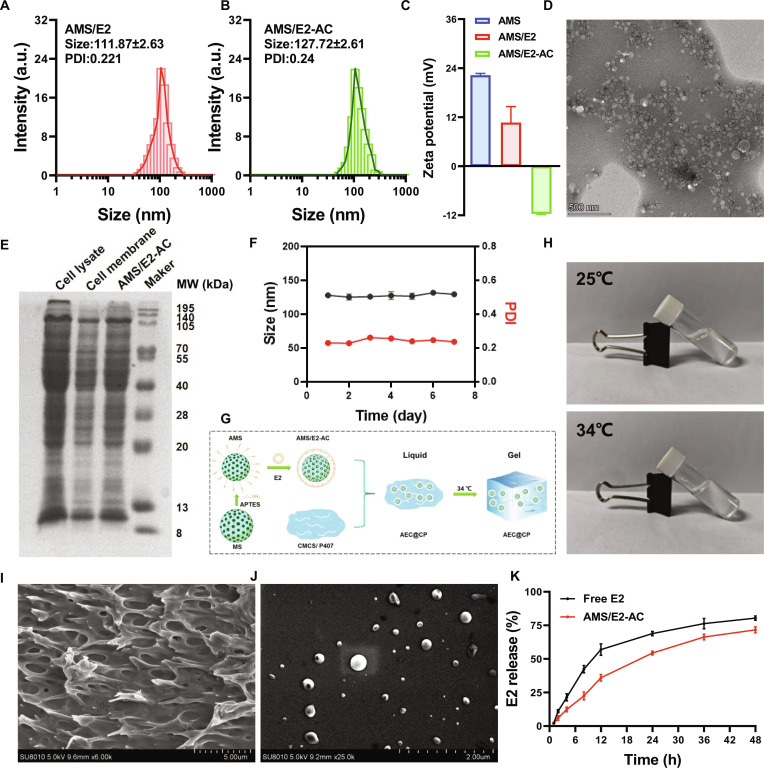
Characterization of the AEC@CP drug delivery system. (A) DLS particle size of AMS/E2 nanoparticles. (B) DLS particle size of AMS/E2-AC nanoparticles. (C) Zeta potentials of AMS, AMS/E2, and AMS/E2-AC. (E) SDS-PAGE results of AMS/E2-AC. (D) TEM image of AMS/E2-AC nanoparticles. (F) Preliminary stability results of AMS/E2-AC over 7 d. (G) Schematic illustration of the preparation of AEC@CP. (H) Thermosensitive changes of AEC@CP. (I) SEM image of AEC@CP (observing the gel’s porous structure). (J) SEM image of AEC@CP (observing nanoparticles within the gel). (K) Transdermal release of E2 results on nasal mucosa.

In order to precisely and effectively deliver E2 to astrocytes, the AEC@CP drug delivery system was constructed. During the preparation of biomimetic nanogel hydrogels, the DLS particle size of AMS/E2 nanoparticles was measured to be (111.87 ± 2.63) nm. After encapsulation with astrocyte membrane, the particle size increased slightly, with the DLS particle size of AMS/E2-AC nanoparticles measured at (127.72 ± 2.61) nm. The zeta potentials of AMS/E2 nanoparticles and AMS/E2-AC nanoparticles are shown in Fig. 5C, with the potential shifting from positive to negative after encapsulation with the astrocyte membrane, further indicating the successful synthesis of AMS/E2-AC. The microscopic morphology of AMS/E2-AC nanoparticles was observed using TEM, as shown in Fig. 5D, where the astrocyte membrane was successfully coated on the surface of AMS/E2 nanoparticles, with a size around 100 nm, slightly lower than the DLS particle size. This difference may be attributed to the fact that TEM measures the dry state particle size, while DLS measures the hydrated particle size. Furthermore, SDS-PAGE results indicate that the astrocyte membrane and AMS/E2-AC have similar protein compositions compared to astrocyte lysates, suggesting successful encapsulation of the astrocyte membrane on the surface of AMS/E2 (Fig. 5E). The stability of AMS/E2 and AMS/E2-AC was assessed by measuring water and particle size over 7 d, as shown in Fig. 5F, demonstrating no significant changes in particle size within 7 d, indicating good stability of both AMS/E2 and AMS/E2-AC. The encapsulation efficiency and drug loading of E2 in AMS/E2-AC were determined by HPLC, with an encapsulation efficiency of (85.20 ± 1.67)% and a drug loading of (6.38 ± 0.12)%. Subsequently, the AEC@CP hydrogel was prepared and its thermosensitive properties were observed, as shown in Fig. 5G, where the AEC@CP thermosensitive hydrogel exhibited a flowable state at 25 °C and gelled at 34 °C, demonstrating good thermosensitive characteristics (Fig. 5H). SEM images of the AEC@CP gel depicted the porous structure of the gel (Fig. 5I) and the encapsulated AMS/E2-AC nanoparticles within the gel (Fig. 5J). The transdermal release behavior of the AEC@CP gel was further evaluated, as shown in Fig. 5K, indicating that the transdermal rate of E2 from AMS/E2-AC could reach (71.64 ± 1.87)% over 48 h, with a slower release behavior compared to free E2, suggesting that AMS/E2-AC extended the overall drug release duration.

### AEC@CP alleviates astrocytic pyroptosis

**Fig. 6.**
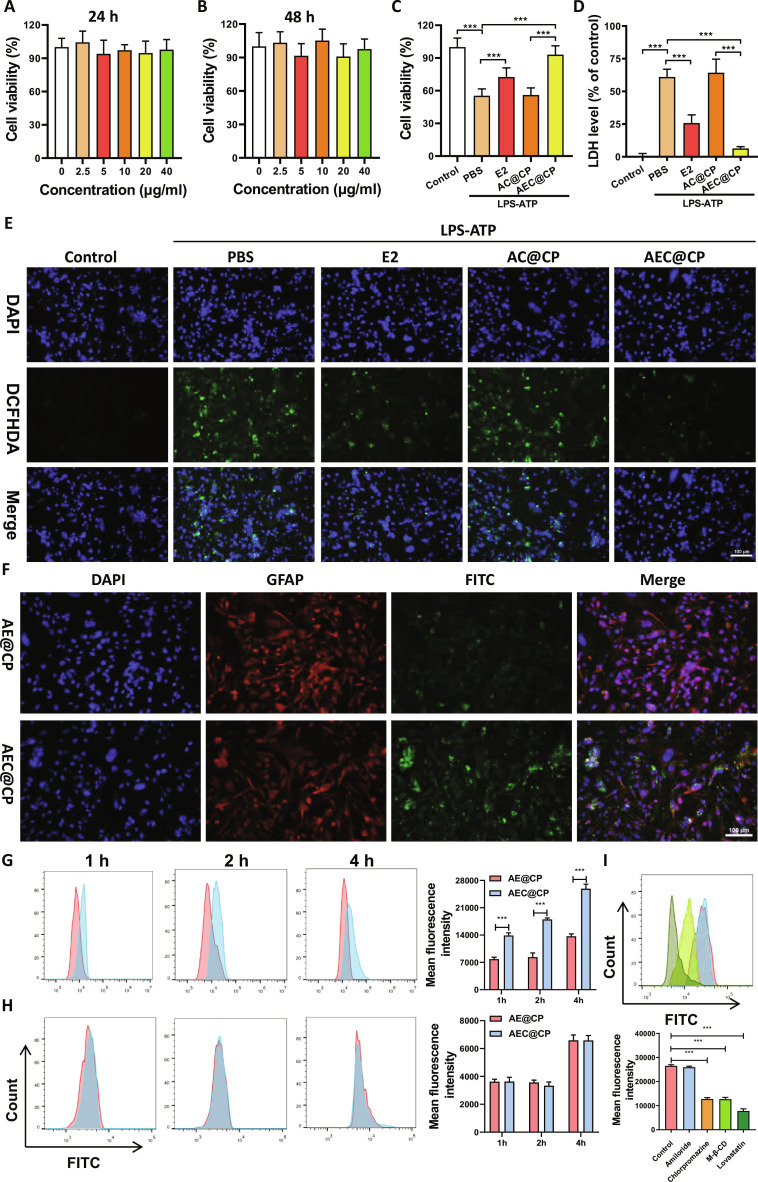
Effect of AEC@CP on astrocytes. (A and B) CCK-8 assay to evaluate the cytotoxicity of different concentrations (0, 2.5, 5, 10, 20, and 40 μg/ml) of empty carriers (AC@CP). (C) CCK-8 assay to assess the impact of AEC@CP on the viability of depressive astrocytes. (D) Assay kit to measure the influence of AEC@CP on LDH release in astrocytes. (E) Immunofluorescence assessing the effect of AEC@CP on ROS production in astrocytes. (F) Immunofluorescence detecting the uptake of AEC@CP by astrocytes at 4 h. (G) Flow cytometry analysis of AE@CP and AEC@CP uptake by astrocytes at different time points. (H) Flow cytometry analysis of AE@CP and AEC@CP uptake by neurons at different time points. (I) Assessment of endocytic pathway dependence using various endocytosis inhibitors in combination with flow cytometry. *P < 0.05, **P < 0.01, ***P < 0.001.

Astrocytic cytotoxicity was evaluated by stimulating astrocytes with empty carriers (AC@CP). Results in Fig. 6A and B demonstrate that the cell viability of astrocytes incubated with different concentrations of AC@CP for 24 and 48 h was above 80%, indicating low cytotoxicity. In vitro, LPS-ATP treatment was used to induce pyroptosis in astrocytes. CCK-8 assay results in Fig. 6C reveal a significant decrease in astrocyte viability in the LPS-ATP group compared to the Control group. However, after treatment with E2 and AEC@CP (both at a concentration of 10 nM), cell viability was notably enhanced. Notably, the viability of astrocytes in the AEC@CP group was significantly higher than that in the E2 group. LDH release results in Fig. 6D show that both E2 and AEC@CP treatments significantly reduce LPS-ATP-induced LDH release. AEC@CP exhibited a stronger inhibitory effect on LDH release compared to E2, indicating that AEC@CP can effectively suppress pyroptosis in depressive astrocytes. Consistent with previous research findings, the fluorescence results in Fig. 6E demonstrate that AEC@CP effectively inhibits the generation of ROS in LPS-ATP-induced astrocytes. Fluorescein isothiocyanate (FITC)-labeled E2 was used to observe the uptake of AE@CP and AEC@CP by astrocytes. As shown in Fig. 6F, due to the encapsulation with astrocyte membrane, AEC@CP can be more efficiently taken up by astrocytes. To further validate the functional role of astrocyte membrane coating, flow cytometry-based cellular uptake studies were conducted. Both AE@CP and AEC@CP were internalized by astrocytes in a time-dependent manner (Fig. 6G). Notably, AEC@CP exhibited significantly higher cellular uptake than AE@CP at all examined time points. In contrast, no significant difference in uptake was observed between AE@CP and AEC@CP in neuronal cells (Fig. 6H), indicating that the enhanced internalization of AEC@CP is astrocyte-specific and mediated by homologous membrane recognition rather than nonspecific uptake. Endocytosis inhibition studies further demonstrated that chlorpromazine, M-β-CD, and lovastatin significantly suppressed the uptake of AEC@CP by astrocytes, whereas amiloride exerted only a minor effect (Fig. 6I). These findings indicate that AEC@CP internalization predominantly occurs through clathrin-mediated endocytosis and lipid raft-dependent pathways, including caveolae-mediated endocytosis.

### AEC@CP efficiently targets mouse brain astrocytes

**Fig. 7.**
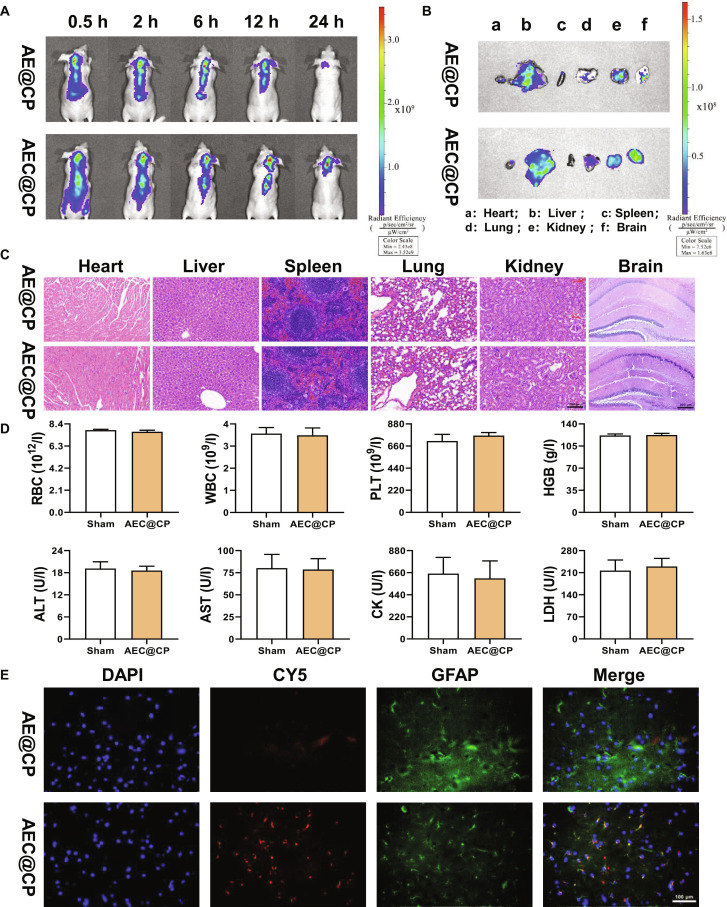
Characterization of the AEC@CP drug delivery system. (A) In vivo distribution of AE@CP and AEC@CP at 0.5, 2, 6, 12, and 24 h after nasal administration. (B) Distribution of AE@CP and AEC@CP in the mouse heart, liver, spleen, lungs, kidneys, and brain after 24 h of nasal administration. (C) Hematoxylin and eosin (H&E) staining of major organs (heart, liver, spleen, lung, kidney, and brain) from treated groups. (D) Biochemical analysis of systemic toxicity markers in different treatment groups. (E) Immunofluorescence detection of the colocalization relationship between AEC@CP and mouse hippocampal astrocytes.

After nasal administration for 24 h, the in vivo distribution of AE@CP and AEC@CP is shown in Fig. 7A. Following nasal administration, both AE@CP and AEC@CP successfully crossed the blood–brain barrier. However, compared to AE@CP, AEC@CP exhibits longer targeted retention in the brain. The distribution of drugs in the heart, liver, spleen, lungs, kidneys, and brain tissues after nasal administration for 24 h is illustrated in Fig. 7B. After 24 h, AEC@CP still shows a strong fluorescent signal in the mouse brain, indicating its prolonged presence in the brain. H&E staining of the heart, liver, spleen, lungs, kidneys, and brain revealed no apparent histopathological abnormalities in the AEC@CP group compared with the AE@CP group (Fig. 7C), indicating good tissue compatibility. Moreover, serum biochemical analysis demonstrated comparable levels of ALT, AST, TG, CHO, BUN, and CK between the 2 groups (Fig. 7D), suggesting that AEC@CP did not induce detectable systemic toxicity. Subsequently, we further investigated the colocalization relationship between AEC@CP and astrocytes using fluorescence microscopy. It was observed that AEC@CP encapsulated with astrocyte membrane exhibited a stronger colocalization with astrocytes, indicating more efficient uptake by astrocytes (Fig. 7E).

### AEC@CP alleviates depressive behavior in perimenopausal depressive mice

**Fig. 8.**
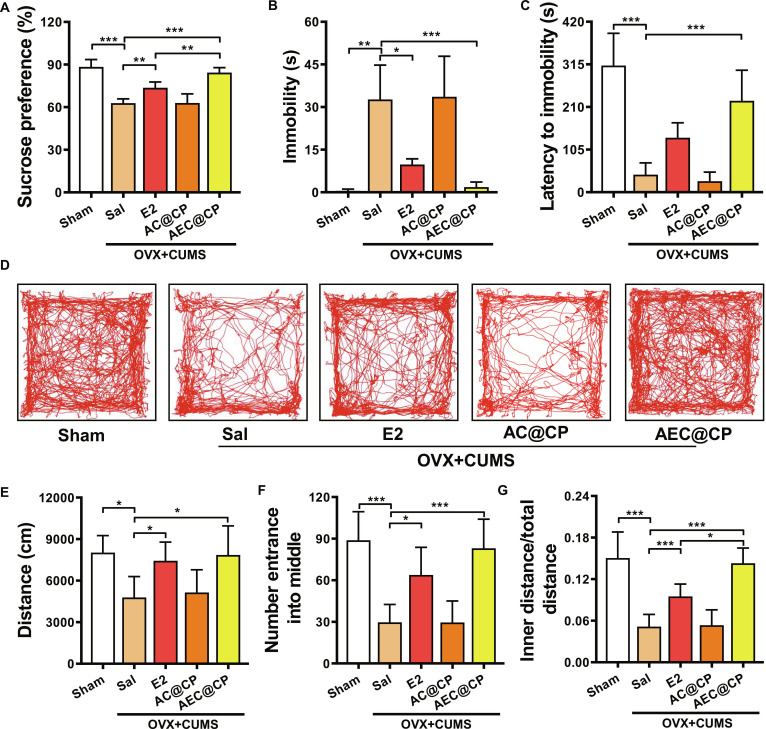
Effect of AEC@CP on perimenopausal depressive behavior in mice. (A) Sucrose preference test. (B) Immobility time in the forced swimming test. (C) Time taken to reach immobility in the forced swimming test. (D) Movement trajectory of mice in the open field test. (E) Total distance traveled by mice in the open field test. (F) Number of entries into the central area by mice in the open field test. (G) Ratio of distance traveled in the central area to the total distance traveled by mice in the open field test. *P < 0.05, **P < 0.01, ***P < 0.001.

A perimenopausal depressive mouse model was established to assess the impact of AEC@CP on depressive behavior in mice. Results in Fig. 8A demonstrate that after treatment with AEC@CP, the sucrose preference of perimenopausal depressive mice was restored to the level of the Sham group, indicating a significant improvement in depressive behavior by AEC@CP. The forced swimming test results, as shown in Fig. 8B and C, reveal that compared to E2, after treatment with AEC@CP, the immobility time of mice significantly decreased, and the time taken to reach immobility also notably increased, indicating further improvement in depressive behavior and the speed of onset. Results of the open field test, displayed in Fig. 8D to G, show that after AEC@CP treatment, the number of entries into the central area, the ratio of distance traveled in the central area to the total distance traveled, and the total distance traveled by mice significantly increased, suggesting an improvement in depressive behavior.

### AEC@CP alleviates hippocampal astrocyte pyroptosis in perimenopausal depressive model mice

**Fig. 9.**
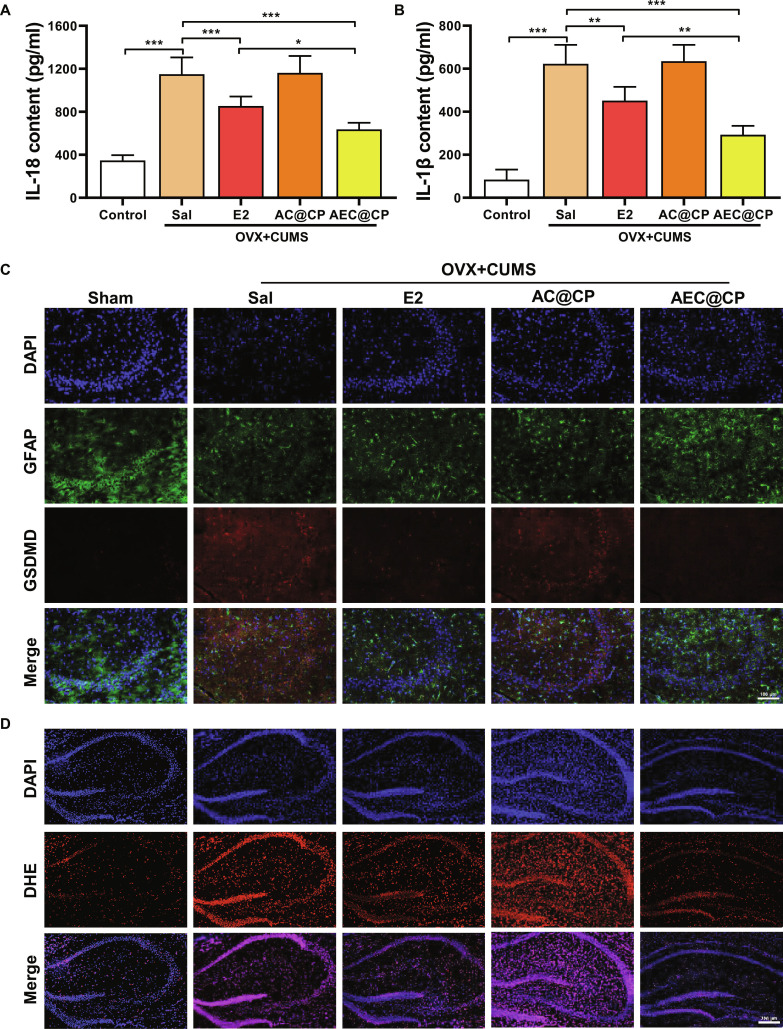
The effect of AEC@CP on hippocampal astrocyte pyroptosis in perimenopausal depressive model mice. (A and B) ELISA experiment to detect the expression of pyroptosis markers IL-18 and IL-1β in the hippocampus. (C) Immunofluorescence investigation of the colocalization relationship between astrocytes and the pyroptosis-related protein GSDMD. (D) DHE staining to measure the ROS levels in the hippocampal tissue.

Further assessment of the effect of AEC@CP on hippocampal tissue pyroptosis in mice was conducted. Results in Fig. 9A and B demonstrate that after treatment with AEC@CP, the expression levels of pyroptosis markers IL-1β and IL-18 significantly decreased, indicating that AEC@CP can notably inhibit the generation of cellular pyroptosis. Immunofluorescence was used to detect the colocalization relationship between hippocampal astrocytes and GSDMD in mouse hippocampal tissue. As shown in Fig. 9C, AEC@CP treatment produced a more pronounced suppression of GSDMD overexpression in the perimenopausal depression model compared with E2 alone, effectively restoring GSDMD expression to levels comparable to those of the Sham group. ROS fluorescence staining was employed to assess the impact of AEC@CP on the oxidative stress levels in mouse hippocampal tissue. As shown in Fig. 9D, AEC@CP treatment led to a more pronounced reduction in ROS generation within the hippocampus compared with E2 alone, effectively mitigating oxidative stress.

## Discussion

In this study, we demonstrated that E2 mitigates perimenopausal depression in mice through the inhibition of astrocytic pyroptosis. Moreover, a biomimetic nanolipogel drug delivery system was developed to achieve targeted delivery of E2 to astrocytes, which markedly enhanced its antidepressant efficacy compared with free E2.

The pathogenesis of perimenopausal depression is currently not fully understood, but recent studies have indicated that pyroptosis plays a crucial role in the process of neuronal death and is associated with many central nervous system diseases, including depression [13,40–43]. Additionally, research has shown a decrease in the number of astrocytes in animal models of depression and in patients with depression [16,17]. Treatment with antidepressants can promote astrocyte proliferation [18,19]. In this study, we observed a decrease in the number of astrocytes and an increase in pyroptosis in astrocytes in perimenopausal depressive mice, providing strong evidence for the significant role of astrocyte pyroptosis in perimenopausal depression. Inhibiting astrocyte pyroptosis may be a promising strategy for treating perimenopausal depression.

Studies have demonstrated that the NLRP3/caspase-1/GSDMD axis-regulated astrocyte pyroptosis plays a key role in mouse models of depression [24]. P2X7R, as an upstream effector of NLRP3, can activate the NLRP3 inflammasome, subsequently activating caspase-1, cleaving GSDMD, and forming the NLRP3–caspase-1–GSDMD signaling axis, leading to cell pyroptosis [44]. In previous research, E2 was found to reverse depression and anxiety-like behaviors induced by OVX, elevated levels of inflammatory markers (IL-1β and IL-18), and increased expression of P2X7R [29]. In this study, we reached a similar conclusion that E2 can improve depressive behavior in perimenopausal mice by inhibiting the expression of P2X7R in astrocytes, suppressing the NLRP3/caspase-1/GSDMD axis, and inducing cell pyroptosis.

To safely and efficiently deliver E2 to the site of disease, a biomimetic nanolipid gel drug delivery system was constructed in this study for targeted delivery of E2 to astrocytes. It has been reported that nanoparticles with naturally cell membrane camouflage, retaining specific proteins and lipids on the cell membrane, can inherit cell functions. For example, coating nanoparticles with red blood cell membranes allows them to evade immune surveillance [32], while encapsulating stem cell membranes around nanoparticles enhances their ability to target tumors [33], and leukocyte membrane camouflage can assist nanoparticles in reaching inflammatory sites [35]. To achieve efficient astrocyte-targeted delivery of E2, astrocyte membranes were coated onto the surface of AMS/E2 nanoparticles to form AMS/E2-AC. In vitro studies demonstrated that astrocyte membrane coating markedly enhanced the time-dependent uptake of nanoparticles by astrocytes, while no preferential uptake was observed in neurons, indicating homologous targeting specificity. Mechanistic analyses further revealed that AEC@CP internalization occurs primarily via clathrin-mediated and lipid raft-associated endocytosis pathways. Notably, the slightly reduced cumulative release of AMS/E2-AC compared with free E2 reflects an intrinsic feature of nanocarrier-based sustained delivery. Kinetic analysis further demonstrated that the release behavior of AMS/E2-AC closely followed the Higuchi model (*Y* = 12.57*X*^1/2^ − 11.00, *R*^2^ = 0.987), confirming a diffusion-controlled mechanism consistent with sustained drug delivery. Moreover, previous studies have consistently reported that drug-loaded nanocarrier systems often exhibit lower cumulative in vitro release than free drugs, which is considered a reasonable and widely accepted phenomenon [45–47].

Furthermore, we encapsulated AMS/E2 nanoparticles in a thermosensitive hydrogel (AEC@CP) for intranasal administration. This mode of administration bypasses the blood–brain barrier, delivering the therapeutic drug directly to the brain via the olfactory and trigeminal nerves, minimizing systemic exposure [48]. It is an excellent therapeutic option for neurological diseases. Injectable thermosensitive hydrogels are promising delivery vehicles, as they are liquid at room temperature and transition to a gel state upon heat stimulation. These viscous hydrogels can adhere to the nasal mucosa, acting as reservoirs for sustained drug release, promoting drug absorption through the nasal epithelium. Due to their porous network structure, hydrogels can effectively load nanocarriers [49]. Taken together, intranasal delivery via olfactory and trigeminal pathways, prolonged nasal residence conferred by the thermosensitive hydrogel, and homologous recognition mediated by the astrocyte membrane coating collectively account for the enhanced brain accumulation and astrocyte-targeting observed in vivo.

In this study, compared to free E2 treatment, AEC@CP demonstrated a stronger ability to combat perimenopausal depressive behavior in mice. It effectively reduced the levels of inflammatory markers IL-1β and IL-18 in astrocytes, and inhibited the P2X7R-mediated NLRP3/caspase-1/GSDMD signaling pathway. Although our current data do not directly elucidate upstream regulatory mechanisms, increasing evidence suggests that estrogen receptor β (ERβ) may play a critical modulatory role. Previous studies have reported that E2 regulation of hippocampal inflammation and depression/anxiety-like behaviors is dependent on ERβ and closely associated with P2X7R expression and inflammasome activation [29]. Similarly, ERβ-mediated down-regulation of P2X7R has been shown to exert anti-inflammatory effects in rat models of inflammatory bowel disease [50]. Therefore, E2 delivered via AEC@CP may activate ERβ in astrocytes, thereby attenuating P2X7R-mediated NLRP3 inflammasome activation and subsequent pyroptotic responses. Future studies employing ERβ knockdown or pharmacological inhibition will be essential to validate this upstream–downstream relationship and further elucidate the molecular basis of AEC@CP-mediated neuroprotection in perimenopausal depression.

In summary, these studies indicate that E2 can improve perimenopausal depressive behavior in mice by inhibiting the P2X7R-mediated NLRP3/caspase-1/GSDMD axis, thus suppressing astrocyte pyroptosis. Additionally, our biomimetic nanolipid gel AEC@CP can deliver E2 to astrocytes, promoting efficient cellular uptake. The therapeutic efficacy of AEC@CP in combating perimenopausal depression is markedly superior to treatment with free E2.

## Ethical Approval

This study was conducted with approval from the Institutional Animal Care and Use Committee at the Zhejiang Center of Laboratory Animals (approval no. ZUCLA-IACUC-20010296).

All animal experiments were carried out in compliance with the guidelines set forth in the 8th edition of the “Guide for the Care and Use of Laboratory Animals”, published by the National Academies Press, National Academy of Sciences, located in Washington, DC.

## Data Availability

The datasets used and/or analyzed during the current study are available from the corresponding author on reasonable request.
