# Supplementary material for: Bionic Nanoparticle Hydrogel for Astrocyte-Targeted Estradiol Delivery Ameliorates Perimenopausal Depression via P2X7 Receptor Inhibition
Source: Biomater Res. 2026 Mar 13;30:0336. doi: 10.34133/bmr.0336 (PMC12982826; doi:10.34133/bmr.0336)
Supplement: Supplementary 1 — Table S1 Figs. S1 and S2 [file bmr.0336.f1.docx]

**Table S1** qRT-PCR primers.

| Genes | Sequence |
| --- | --- |
| NLRP3 | F 5'-CCTCGTCACCATGGGTTCTG-3' |
|  | R 5'-GGCTTAGGTCCACACAGAAAGT-3' |
| GSDMD | F 5'-AGTGCTCCAGAACCAGAACCG-3' |
|  | R 5'-TCTCCCATGCCTGACAACATC-3' |
| P2X7R | F 5'-AAGGCCAAGAAGTTCCAACCTAGA-3' |
|  | R 5'-CCATTGAGAGCATGGCTTCTTG-3' |
| Caspase 1 | F 5'-CCAGGAGGGAATATGTGGGAC-3' |
|  | R 5'-ACTCCTTGTTTCTCTCCACGG-3' |
| GAPDH | F  5′-AAGAGGGATGCTGCCCTTAC-3′ |
|  | R 5′-CCAATACGGCCAAATCCGTTC-3′ |

**
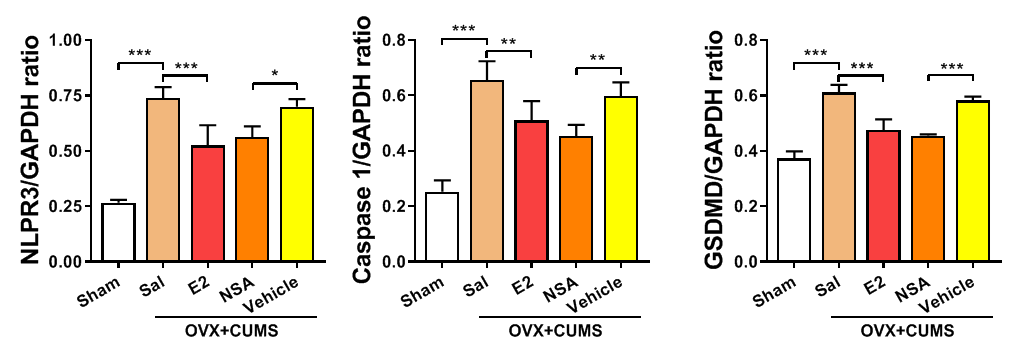
**

**Figure S1.** Western blot analysis of pyroptosis-related protein expression in cells. Protein levels were quantified and normalized to the internal control, and results are presented as relative protein expression.


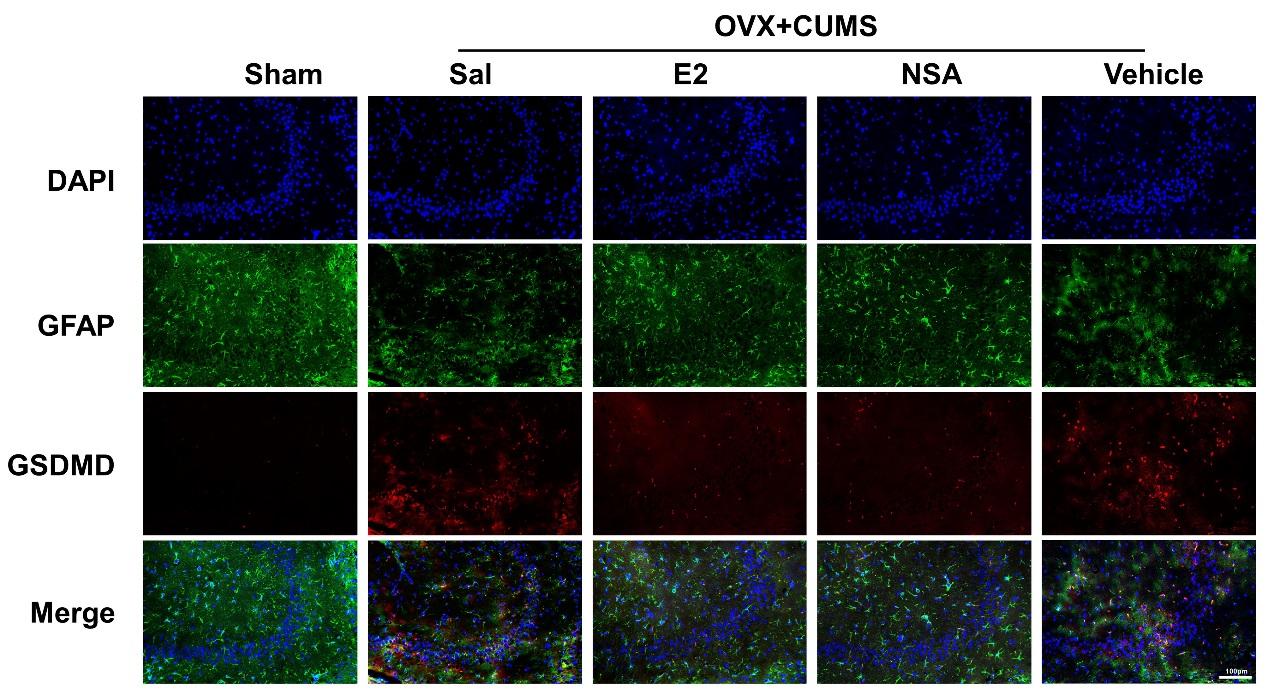


**Figure S2.** Co-localization relationship between astrocytes and pyroptosis-related protein GSDMD detected by immunofluorescence.
